# Comparative transcriptome analysis of *Trichoderma reesei* reveals different gene regulatory networks induced by synthetic mixtures of glucose and β-disaccharide

**DOI:** 10.1186/s40643-021-00411-4

**Published:** 2021-07-03

**Authors:** Yonghao Li, Jingze Yu, Peng Zhang, Tingting Long, Yi Mo, Jianghong Li, Qian Li

**Affiliations:** Chongqing Key Laboratory of Industrial Fermentation Microorganism and School of Chemistry and Chemical Engineering, Chongqing University of Science and Technology, Chongqing, 401331 China

**Keywords:** *Trichoderma reesei*, Cellulase, Transcriptome, Inducer, Transcription factor, Transporter

## Abstract

The mixture of glucose and β-disaccharide (MGD) synthesized by transglycosylation of glucose as a low-cost soluble carbon source can efficiently induce cellulase production in *Trichoderma reesei*, which holds potential for the biorefining of lignocellulosic biomass. However, it is not yet fully understood how MGD induces *T. reesei* cellulase. In this study, transcriptomic analyses were conducted to investigate the molecular basis of MGD for lignocellulose-degrading enzyme production of *T. reesei* Rut C30 compared with that on lactose. Particular attention was paid to CAZymes, transcription factors, transporters and other protein processing pathways related to lignocellulose degradation. As a result, MGD can elicit transcription of GH5-, GH6- and GH7-encoding cellulases that is up to 1.4-fold higher than that induced by lactose, but GH11- and GH74-encoding xylanases are downregulated by 1.7- and 4.4-fold, respectively. Gene expression profiles suggest that the transcription activators *xyr1* and *vib1* are significantly upregulated and that the mitogen-activated protein kinase pathway is strengthened compared to the case of lactose induction. In addition, *hac1-*encoding UPR-specific transcription factors are significantly upregulated by MGD, which may be enhanced due to proper folding and processing of nascent proteins. These findings provide a theoretical basis for further understanding the characterization of efficient cellulase production using MGD as an inducer in *T. reesei* and offer potential strategies for strain improvement.

**Supplementary Information:**

The online version contains supplementary material available at 10.1186/s40643-021-00411-4.

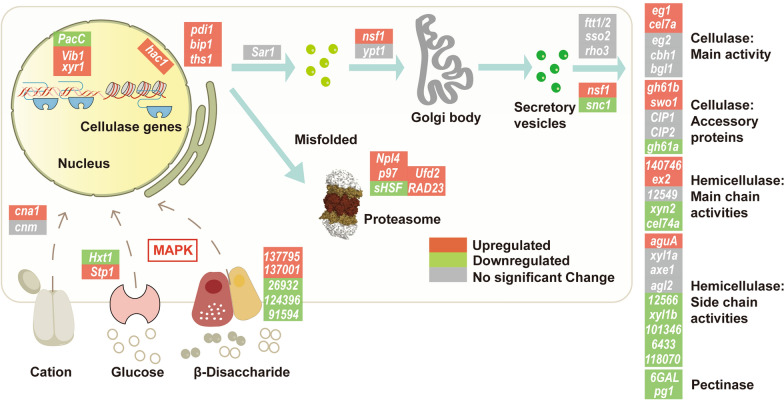

## Introduction

The gradual depletion of fossil fuels and concerns about their environmental influence have diverted much attention to the exploration of renewable and environmentally friendly fuels. Biofuels produced from biorefining of lignocellulosic feedstocks such as corn stover are alternative solutions for these challenges (Langsdorf et al. 2021; Liu et al. 2019; Giri et al. 2020). Lignocellulose predominately consists of cellulose, hemicellulose and lignin that are tightly interlinked, which requires pretreatment and hydrolysis of the corresponding monomers (Rezania et al. 2020; Zhang et al. 2021). The filamentous fungus *Trichoderma reesei* is one major producer of enzymes needed to decompose the polymers above to soluble monosaccharides. In particular, *T. reesei* Rut C30, which is one of the most widely used fungi for the production of cellulolytic enzymes, is very promising for industrial cellulase generation (Bischof et al. 2016).

Cellulase production by *T. reesei* is dependent on the existence of an inducer, which is largely modulated by complicated controlling networks, thus decreasing the productivity of native enzymes (Peterson et al. 2021). As cellulose is a natural inducer of lignocellulase synthesis, the high cost of pure cellulose and low titer productivity when using lignocellulose as an inducer are still challenges. Lactose is preferred as a soluble and inexpensive inducer, but is a less effective cellulase inducer than cellulose. Sophorose is the most efficient inducer of cellulase generation by *T. reesei* (Bischof et al. 2016).

In our previous study, a mixture of glucose–disaccharides (MGD), including sophorose, prepared from glucose via transglycosylation catalyzed by β-glucosidase was validated as an efficient and low-cost inducer. The cellulase titer using MGD as an inducer reached 90.3 FPU/mL, which is drastically higher than that of cellulose or lactose (Li et al. 2016). Moreover, cellulase production with the *T. reesei* mutant reached 102.63 IU/mL using MGD as an inducer (Chen et al. 2020). However, the underlying mechanism of MGD induction is still unclear, particularly compared to the commonly used lactose.

In addition, we tested the biomass hydrolysis capacity of crude cellulases of *T. reesei* induced by MGD. When mixed with commercial cellobiase, the glucose concentration increased to 122.5 g/L by saccharification of corn stover (Li et al. 2017a, b). However, the degradation rate of cellulose is slower than that of commercial enzymes, and the hydrolysis efficiency of hemicellulose is low (Li et al. 2018). To the best of our knowledge, the cellulolytic enzyme system produced by fungal species mainly consists of endoglucanases, cellobiohydrolases and β-glucosidases, and cellulose degradation results from the synergy of these enzyme components. Additionally, auxiliary proteins are critical in the oxidative decomposition of lignocellulosic biomass. Auxiliary proteins include expansin-like swollenin (SWO) and lytic polysaccharide monooxygenases (LPMOs), which were recently reclassified from glycoside hydrolase 61 (GH) to auxiliary activity 9 (Thoresen et al. 2021). To date, there is no study on the effect of this lignocellulose-degrading enzyme on MGD as an inducer by *T. reesei*.

Abundant transcriptomic data have enriched the understanding of cellulase synthesis regulation and led to the discovery of novel genes and transcription factors. This study compares the transcriptomes of *T. reesei* grown on MGD and on lactose, aiming to demonstrate the differences in the enzymes produced and to clarify the molecular basis of lignocellulase production. These findings offer novel insights, such as transporters, transcription factors, the mitogen-activated protein kinase (MAPK) pathway, the ER protein processing pathway and carbohydrate active enzymes, in response to MGD. These results will further increase industrial *T. reesei* strains by metabolic engineering to generate abundant lignocellulose for biorefining of lignocellulose.

## Methods

### Materials, strain and growing conditions

The mixture of glucose–disaccharides (MGD) was synthesized from glucose by β-glucosidase through a transglycosylation reaction. Specifically, β-glucosidase was added to a substrate containing 600 g/L glucose at 20 CBU/g (glucose), and the transglycosylation reaction was performed at 65 °C and pH 4.8 for 72 h, after which the β-glucosidase was deactivated by incubating the mixture at 100 °C for 5 min. Finally, the MGD contained 410.20 g/L glucose, 60.56 g/L gentiobiose, 9.34 g/L cellobiose and 13.66 g/L sophorose (Li et al. 2016).

*Trichoderma reesei* RUT C30 (NRRL 11460) was a gift from the USDA ARS Culture Collection. The strain was grown on a plate containing 3% malt extract and 1.5% agar for 7 d for sporulation. For RNA sequencing or cellulase production, a spore suspension was cultivated in 250-mL Erlenmeyer flasks with 50 mL of medium, including 4 g/L glucose and 10 g/L corn steep liquor. The cultivation proceeded at 28 °C and 150 rpm for 24 h, and then mycelium was cultured at 10% (v/v) in 50 mL of fermentation medium supplemented with 10 g/L MGD or lactose as the sole carbon source. The fermentation medium for cellulase generation by *T. reesei* Rut C30 was formulated from a previous recipe, which contained 10 g/L MGD or 10 g/L lactose, 1 g/L peptone, 0.3 g/L urea, 0.8 g/L CaCl_2_, 0.5 mL/L Tween-80, 4 g/L KH_2_PO_4_, 0.6 g/L MgSO_4_·7H_2_O, 2.8 g/L (NH_4_)_2_SO_4_, 10 mg/L FeSO_4_·7H_2_O, 3.4 mg/L MnSO_4_·H_2_O, 2.8 mg/L ZnSO_4_·7H_2_O, 4 mg/L CoCl_2_ and 500 ml/L 0.2 M Na_2_HPO_4_–citric acid (pH 5.0). After growth on MGD or lactose for 36 or 48 h, the cultures were centrifuged for harvest of mycelia, which were immediately frozen and kept at −80 °C until RNA isolation.

### RNA isolation and high-throughput RNA-seq

Total RNA was isolated using a Spin Column Plant total RNA purification kit (Sangon Biotech, China) as instructed by the manufacturer. Two repeated assays and RNA-seq were performed. A 2100 Bioanalyzer (Agilent) was used for RNA quantification and integrity examination. An Agilent 2100 bioanalyzer (Agilent RNA 6000 Nano Kit) was applied for total RNA sample QC, including the RNA content, 28S/18S, RIN and fragment length distribution. RNA purity was identified by NanoDropTM.

The RNA-seq libraries were sequenced on an Illumina HiSeq 4000 system, followed by quality filtration and mapping to the *T. reesei* RUT C30 reference genome (JGI Genome Portal, http://genome.jgi-psf.org/Trire2/Trire2.home.html). Clean reads were mapped to references using Bowtie2, and then the gene expression level was calculated with RSEM. RSEM is a software package for estimating gene and isoform expression levels from RNA-Seq data. The Pearson correlation between different samples is an important parameter to test the reliability of the experiment and whether the sample selection is reasonable. Then, we calculated the Pearson correlation between all samples using cor, performed hierarchical clustering between all samples using hclust, and drew diagrams with ggplot2 with R functions. Differentially expressed genes were analyzed using DEseq2, which is based on the negative binomial distribution, performed as described in previous research (Love et al. 2014), and the thresholds were a two-time change cutoff (log_2_-fold change) ≥ 1 or ≤ −1 and an adjusted *p* ≤ 0.05. The filter statistic in DESeq2 is the mean of normalized counts for a gene, while the test statistic is *p*, the *P* value from the Wald test. Genes were divided by the major annotation in the Gene Ontology (GO) and EuKaryotic Orthologous Groups (KOG) systems (*T. reesei* RUT C30 genome database v1.0). The data presented in this study are deposited in the National Center for Biotechnology Information (NCBI). The website link is https://www.ncbi.nlm.nih.gov/sra/PRJNA714230.

### RT-qPCR

To verify the RNA-seq data, 23 genes, including hydrolytic enzymes, were further investigated by RT-qPCR using SYBR® Premix Ex Taq™ II (TaKaRa, Japan) and a CFX connect™ real-time system (Bio-Rad, USA). PCR was set up as per the manufacturer’s manual. Relative transcription of genes was normalized by 2^−ΔCt^, with *sar1* as an endogenous control. The same RT-qPCR method was used to analyze the expression level of the cellulase gene during the fermentation process. The primers are listed in Additional file 3: Table S1. Pearson correlation was calculated using GraphPad Prism v8 software (Graphpad Software Inc., La Jolla, CA).

### Analytical methods

Total reducing sugar was analyzed by the dinitrosalicylic acid (DNS) method recommended by NREL (Miller 1959). The concentration of glucose was determined using an M-100 biological sensor (Shenzhen Sieman Technology Co., Ltd., Shenzhen, China). Cellulase activity (FPA) was determined using filter paper (No. 1, Whatman) as recommended (Ghose 1987). One unit (U) of enzyme activity is defined as the amount of enzyme that produces 1 μmol of reducing sugar as glucose in the reaction mixture per minute. The concentrations of secreted proteins were determined using Bradford reagent (Sangon Biotech, China).

## Results and discussion

### Comparison of the MGD- and lactose-regulated transcriptome

**Fig. 1:**
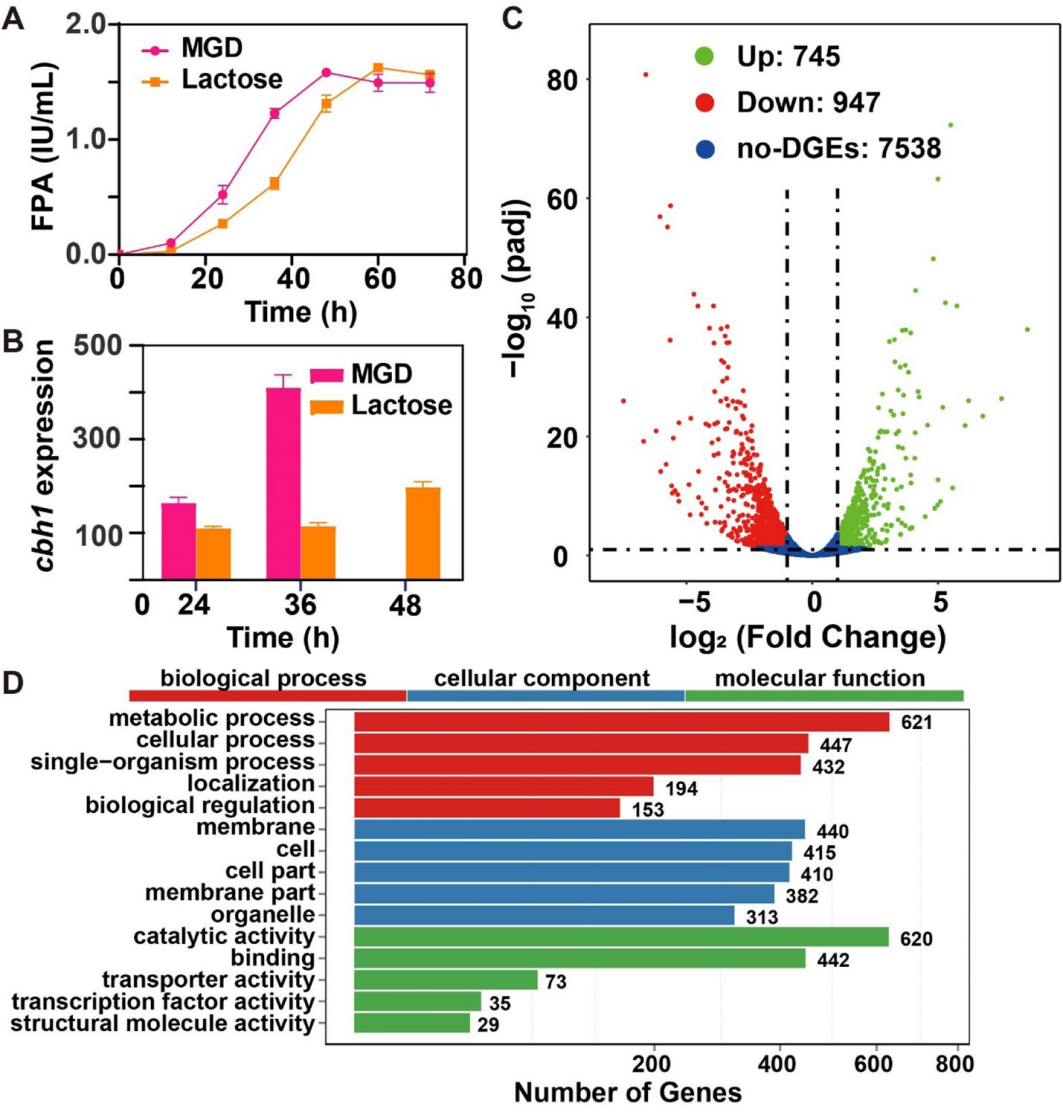
Comparison of cellulase production (**A**) and transcription of *cbh1* encoding cellulase (**B**) in the batch fermentation of the strain *T. reesei* Rut C30 using 10 g/L MGD or 10 g/L lactose as the inducer and carbon source. **C** The volcano map shows the comparison of gene expression levels. Green dots represent genes that were significantly upregulated; red dots represent genes that were significantly downregulated; blue dots represent genes for which no significant differences were observed. **D** Gene Ontology (GO) enrichment analysis of *T. reesei* DEGs in MGD and lactose. Significantly enriched categories (*P*adj < 0.05) are shown

*Trichoderma reesei* Rut C30 was cultured directly in a mixture of glucose–disaccharides (MGD) or lactose as a carbon source or inducer. Previously, we demonstrated its growth, carbon source consumption, cellulase/xylanase production and several main cellulase/xylanase gene expression profiles on MGD or lactose (Li et al. 2016), and similar results were shown in this study. Additional file 1: Fig. S1A and B show that the utilization of MGD was faster than lactose in *T. reesei*, and the highest protein concentration and cellulase yield were achieved at 48 h in MGD compared to 60 h in lactose (Additional file 1: Fig. S1C and Fig. 1A). In addition, the expression of the main cellulase gene (*Cbh1*) was enhanced as cells grew, with peak expression detected at approximately 36 h. However, lactose, as an inducer, stimulated the peak expression of the cellulase gene in *T. reesei* at 48 h (Fig. 1B). Based on these data and to compare the expression of genes initiated in *T. reesei* on two soluble inducers, we determined the transcriptional profiles during the highest expression of the cellulase gene. In our project, we sequenced 4 samples, which generated in total approximately 4.45 Gb per sample on the Illumina HiSeq Platform. The sequencing reads contain low-quality, adaptor-contaminated and abundant unknown base (N) reads, which must be removed before downstream investigations. The quality metrics of clean reads can be found in Additional file 4: Table S2. Next, the clean reads (91.65% reads on average) were mapped to the reference genome using HISAT. The mapping results are uniform (Additional file 5: Table S3), suggesting that the samples are comparable. The samples used in the transcriptional analysis were highly correlated (Pearson correlation, *r*^2^ ≥ 0.978, Additional file 2: Fig. S2A). Based on the expression information, the boxplots (Additional file 2: Fig. S2B) and density map (Additional file 2: Fig. S2C) show that the profiles of normalized samples.

Based on gene expression, we identified the differentially expressed genes (DEGs) between samples or groups. We used DEseq2 to identify 1692 DEGs (adjusted *P* ≤ 0.05) in MGD/lactose cells (Fig. 1A; Additional file 6: Table S4). At log_2_-fold change ≥ 1 or ≤  − 1, 745 and 947 genes were up- and downregulated exclusively on MGD/lactose (Fig. 1B). GO annotation of the 1692 carbon source-specific genes revealed that cellular metabolism was more active with MGD induction than with lactose, since the functions of DEGs were mainly related to carbohydrate metabolism, cell membrane, and cellular processes, and there were significant changes in molecular functions including catalytic activity, binding and transport activities (Fig. 1C). These results highlight the possibility of discovering genes participating in cell growth and cellulase generation in *T. reesei* under MGD or lactose as inducers and carbon sources. However, cell modulation and cellulase production by *T. reesei* are extremely complex processes that need further analysis.

To further assess the carbon source-specific regulons (Fig. 1B), we identified the top 10 genes that were exclusively up- or downregulated on MGD/lactose (Table 1). The top 10 upregulated genes using MGD as an inducer included glycoside hydrolase 16 (GH16) (TrireC30_111701), and the top 10 downregulated genes included GH28 (TrireC30_133383) and GH54 (TrireC30_102517) (Table 1). Interestingly, the top 10 upregulated genes in lactose involve more GHs than in MGD, even if the ingredients of MGD are more complicated. The other upregulated genes in MGD contain three regulating cell wall proteins (TrireC30_37721, 97585 and 45199), two short chain dehydrogenase proteins (TrireC30_88601), a Ca^2+^-modulated nonselective cation channel polycystin (TrireC30_99737) and three proteins of unknown function. Studies show that Ca^2+^ has an active effect on the cellulase production of *T. reesei* and can regulate the transcription factor *Crz1*, which binds to the *Xyr1* promoter region to regulate cellulase gene transcription (Chen et al. 2016, 2018). However, the transcription of *Crz1* is not significantly upregulated, so the effect of TrireC30_99737 on cellulase production needs to be confirmed by experiments in the future. Other upregulated genes seem to be more conducive to strain growth in MGD than in lactose.

**Table 1:** Log_2_-fold change (FC) of the top 10 genes differentially expressed in MGD/lactose

Condition	Gene ID	Annotation	FC
Up	140199	Predicted protein	8.55
	37721	Cell wall protein	7.52
	112481	Predicted protein	6.78
	88601	Short chain dehydrogenase	6.21
	99737	Ca^2+^-modulated nonselective cation channel polycystin	6.07
	111701	Glycoside hydrolase family 16	5.75
	80523	Predicted protein	5.57
	97585	Cell wall protein	5.50
	45199	Cell wall protein	5.29
	92192	Short-chain dehydrogenase	5.18
Down	34878	Serine carboxypeptidases	− 7.50
	89390	Short-chain dehydrogenase	− 6.71
	133383	Glycoside hydrolase family 28	− 6.62
	73059	Predicted protein	− 6.21
	102517	Glycoside hydrolase family 54	− 6.06
	84969	Serine/threonine protein kinase	− 6.02
	134355	Predicted protein	− 5.81
	26932	MFS sugar transporter	− 5.76
	68550	Kynurenine 3-monooxygenase and related flavoprotein monooxygenases	− 5.65
	124396	MFS sugar transporter	− 5.63

The top 10 downregulated genes included serine carboxypeptidases (TrireC30_34878), short chain dehydrogenase (TrireC30_89390), serine/threonine protein kinase (TrireC30_84969), kynurenine 3-monooxygenase (TrireC30_68550), two MFS permeases (TrireC30_26932 and 124396) and proteins with unknown function (Table 1). The reduced expression of serine carboxypeptidases may be more conducive to the stability of lignocellulosic enzymes, and it is a potential target gene for increasing cellulase production. Moreover, it is noteworthy that there are two genes belonging to MFS families in which the transcription levels are 54 and 50 times higher when induced by lactose than by MGD. These genes are potential lactose transporters or may play a key role in lactose sensing if lactose acts as the true inducer of cellulase secretion. Our results indicate that the expression of lignocellulase and other genes changes in response to the inducer or carbon source available to *T. reesei* (Antoniêto et al. 2014; Castro et al. 2016).

### GH family

**Fig. 2:**
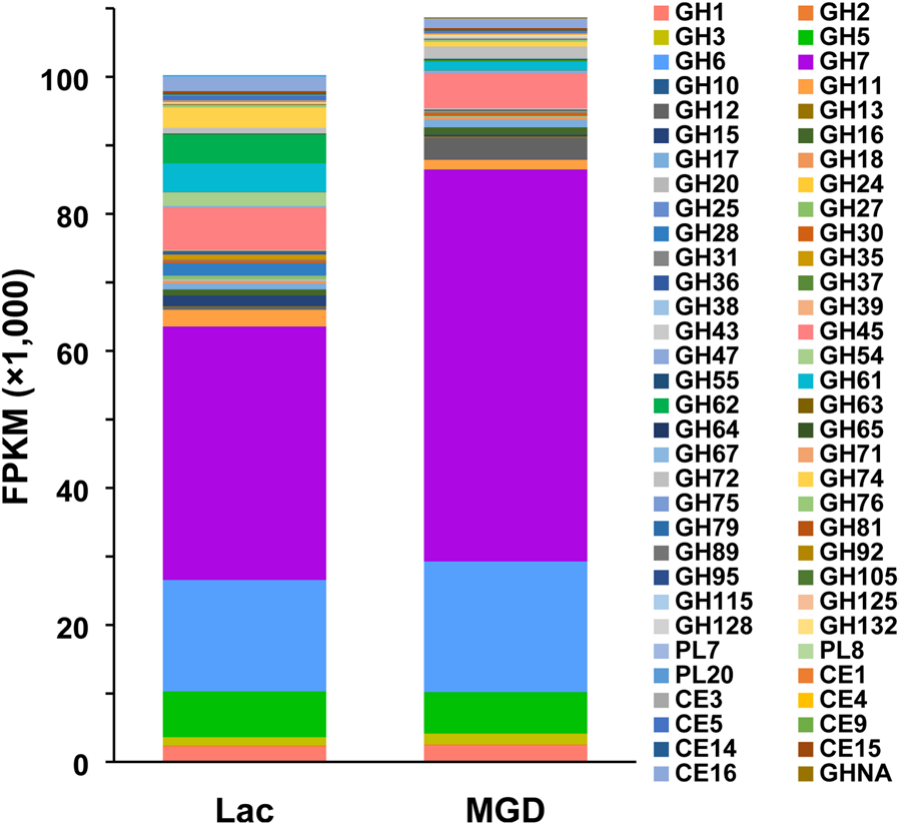
Glycolic hydrase (GH) family (CAZy) genes and expression data from transcriptome analysis. The means of the fragments per kilobase of transcripts per million mapped reads (FPKM) for each glycolic hydrase (GH) family when cultured in lactose (Lac) and MGD (mixture of glucose and disaccharide)

To comprehensively analyze the transcriptional differences in lignocellulosic degrading enzyme genes of *T. reesei* using MGD or lactose as an inducer, we analyzed the differences in all annotated glycoside hydrolase genes (Häkkinen et al. 2012). The average FPKM for all the genes of a single glycoside hydrolase family was computed. The sum of all the FPKM means for each GH family when incubated in MGD or lactose reflects the overall enzymatic potential and global transcriptional response (Fig. 2; Additional file 7: Table S5). GH5, GH6 and GH7 account for the highest proportion and encode the main endonuclease and exonuclease cellulases (Castro et al. 2014). The transcription of these three GH family members accounts for 59.8% of the glycoside hydrolase activity under lactose as an inducer, whereas growth with MGD considerably induces 75.7%. While MGD can provoke higher levels of cellulase gene expression (Li et al. 2016), the transcription of GH5-, GH6- and GH7-encoding major cellulases is 1.4-fold higher than that induced by lactose. In addition, the expression of GH11 and GH74, encoding a major xylanase, is downregulated by 1.7- and 4.4-fold, respectively (Karimi et al. 2016). Other GH family members present in large proportions include GH1, GH3, GH11, GH12, GH16, GH17, GH45, GH61, GH72 and GH74.

In detail, Table 2 shows the main CAZymes that are differentially expressed in MGD and lactose. Interestingly, the genes of enzymes involved in side chain activities of hemicellulase, such as xylanases (TrireC30_139631, 101346, 6433, 118070 and 102517) and pectinase (TrireC30_11580 and 133383), tend to be induced by lactose but not MGD. Auxiliary activity protein (AA) is not directly involved in cellulose degradation but can assist cellulase in accelerating the degradation rate (Corrêa et al. 2016). More studies have focused on LPMOs that can oxidize β-1,4 glycosidic bonds so that the lignocellulosic chains can be opened to reduce crystallinity and improve hydrolysis efficiency (Brenelli et al. 2016). Furthermore, auxiliary activity 9 (TrireC30_122518) was upregulated in MGD, consistent with a function in cellulose oxidation, but the other AA9 gene (TrireC30_139633) was downregulated. Follow-up experiments should increase gene expression to strengthen cellulose degradation through genetic engineering. The expansion device factor *Swo1* can destroy the structure between lignin and hemicellulose so that more cellulose can be exposed for hydrolyzation by cellulase (Eibinger et al. 2016). In addition, the fact that *Cip1* and *Cip2* can speed up lignocellulosic hydrolysis was verified (Lehmann et al. 2016). With MGD as an inducer, the expression of the *Swo1* gene was higher than that with lactose, but the *Cip1* and *Cip2* gene expression was not significantly different. In conclusion, the gene transcription level of lignocellulase can be regulated by different inducers, so the powerful hydrolysis ability of *T. reesei* lignocellulase can be increased by genetic engineering or inducer mixtures (Li et al. 2017a, b), which may be a solution to the low secretion of xylanase and auxiliary activity by *T. reesei* using MGD as the inducer.

**Table 2:** Log_2_-fold change (FC) of the main secreted lignocellulases expressed in MGD/lactose

Category	Protein id	Name	FC
Cellulase: main activity	5304	Endoglucanase 1	1.55
	12525	Exoglucanase 1	0.89
	72489	Endoglucanase 2	NS
	122470	Exoglucanase 2	NS
	136547	β-glucosidase 1	NS
Cellulase: accessory proteins	122518	Endoglucanase 7 (AA9)	1.00
	104220	Swollenin	0.76
	121449	Cellulose binding domain CIP1	NS
	125575	Cellulose binding domain CIP2	NS
	139633	Endoglucanase 4 (AA9)	− 2.13
Hemicellulase: main chain activities	140746	β-xylosidase	1.16
	124931	Endoxylanase	1.11
	12549	β-mannosidase	NS
	38418	Endo-1,4-β-xylanase	− 1.97
	111943	Xyloglucanase	− 2.13
Hemicellulase: side chain activities	90302	α-glucuronidase	1.48
	72252	α-l-arabinofuranosidase B	NS
	136770	Acetyl esterase	NS
	139631	Acetylxylan esterase	− 1.73
	12566	α-galactosidase 2	NS
	101346	β-galactosidase	− 2.14
	6433	α-d-galactosidase	− 3.16
	118070	α-N-arabinofuranosidase 2	− 4.55
	102517	α-l-Arabinofuranosidase B	− 6.06
Pectinase	11580	Endo-β-1,6-galactanase	− 3.52
	133383	Endopolygalacturonase	− 6.62
Chitinase	99285	β-N-acetylhexosaminidase	NS

NS, Nonsignificant at *P* > 0.05

### Transcription factors

In this analysis, 493 genes in the *T. reesei* Rut C30 genome were predicted to function as transcription factors (TFs) (Castro et al. 2014). The expression of TF-encoding genes in *T. reesei* already known to modulate lignocellulase expression is shown in Table 3. Only the *Xyr1*, *Vib1* and *PacC* genes showed significant differences in MGD compared with lactose (*P*adj < 0.05), indicating similar induction modes of the two inducers to some extent. *Xyr1* and *Vib1* were significantly upregulated by 1.63- and 4.56-fold, respectively, which may explain why MGD has a stronger induction effect on cellulase. In previous studies, *Xyr1* was considered to be essential for cellulase expression in all *T. reesei* strains (Cao et al. 2019). Moreover, the transcription level of *Vib1* influences cellulase production in *T. reesei* Rut C30 (Zhang et al. 2016), and cellulase production and protein secretion were significantly improved by 200% and 219%, respectively, by overexpressing *Vib-1* in *T. reesei* Rut C30 (Zhang et al. 2018). These results support the assumption that *Xyr1* and *Vib1* are important positive controllers of cellulase gene expression (Stricker et al. 2006; Zhang et al. 2017; Xiong et al. 2014). In addition, the expression of *PacC* (pH-responsive TF) decreased by 44.7%. It was modulated in a carbon source-dependent manner and was upregulated under the induction of cellulose (Tilburn et al. 1995). This may be another reason for the high expression of cellulase genes in MGD.

**Table 3:** Log_2_-fold change (FC) of characterized transcription factors involved in the regulation of lignocellulase genes

Gene ID	Name	Log_2_FC	Up/down	Positive/negative-acting
98788	XYR1	0.71	Up	Positive
122363	ACE1	NS		Negative
32395	ACE2	NS		Positive
93466	HAP2	NS		Positive
24298	HAP3	NS		Positive
95791	PacC	− 0.85	Down	Negative
140814	AreA	NS		Positive
98455	ACE3	NS		Positive
91236	BglR	NS		Positive
68701	CLR-1	NS		Positive
76250	CLR-2	NS		Positive
125610	Vib1	2.28	Up	Positive

NS, nonsignificant at *P*adj > 0.05; Lac: lactose

**Fig. 3:**
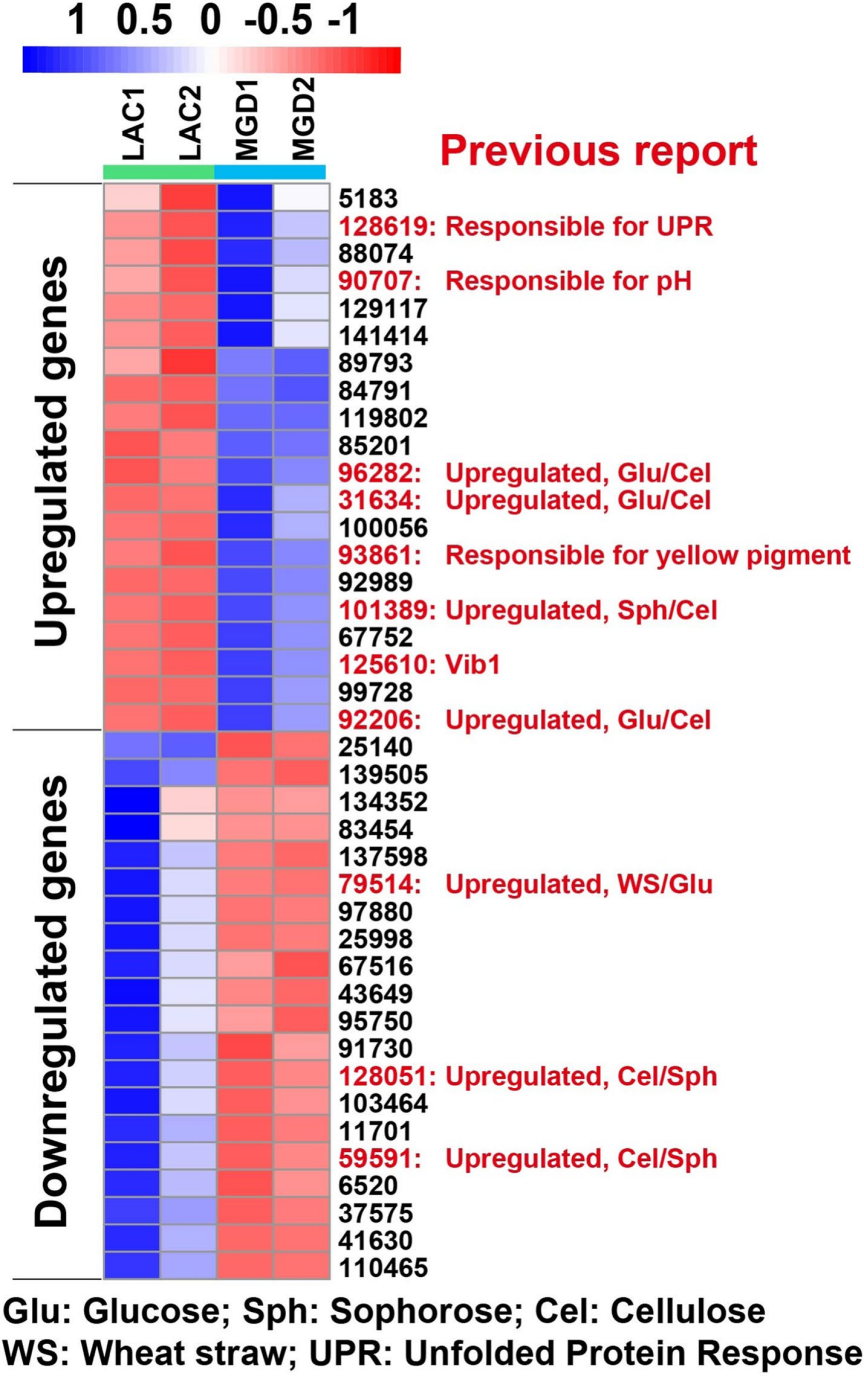
The top 40 genes of transcription factors that are differentially expressed in MGD or lactose. The columns for LAC1 and LAC2 show the gene expression data using 10 g/L lactose as an inducer at 48 h. The columns for MGD1 and MGD2 show the gene expression data using 10 g/L MGD as an inducer at 36 h. Data are shown as a heat map with the following color code of relative expression values (navy: 1; white: 0; firebrick: − 1; numbers indicate the log_2_ of the relative expression level). The previously reported transcription factors were explained

Transcriptome data showed that 45 TF-encoding genes were upregulated and 34 TFs were downregulated in MGD (Additional file 8: Table S6). The 40 TFs with the most significant changes were further analyzed (Fig. 3). Several TFs in this group were reported in other studies. Among the 20 upregulated TFs, TrireC30_101389 (Snd1/p100) was upregulated when induced by sophorose compared with cellulose, and TrireC30_92206, TrireC30_93861, TrireC30_31634 and TrireC30_96282 were upregulated in glucose compared with sophorose and cellulose (Castro et al. 2014). Among those, TrireC30_93861 was identified as the main regulator responsible for yellow pigment synthesis (Derntl et al. 2016), which may be related to the obvious increase in yellow pigment secretion induced by MGD. In addition, *Vib1* (TrireC30_125610) was proven to be responsive to nitrogen and carbon starvation, and the yield of cellulase increased by onefold with the overexpression of this gene (Gao et al. 2017). TrireC30_90707 is related to pH, and reportedly, the transcription of TrireC30_90707 in medium at low pH (3.0) is significantly higher than that at pH 6.0, which leads to decreased cellulase production during TrireC30_90707 overexpression (Häkkinen et al. 2015). *Hac1* (Trirec30_128619), a specific TF that regulates the unfolded protein response (UPR) (Carvalho et al. 2012; Saloheimo et al. 2003), is significantly upregulated, indicating that the UPR is opened under induction by MGD.

Among the top 20 downregulated TFs, it was reported that the transcription levels of Trirec30_128051 and Trirec30_59591 induced by cellulose were significantly higher than those induced by sophorose and glucose (Castro et al. 2014), and Trirec30_79514 expression was higher in wheat straw than in glucose (Bischof et al. 2013). These results preliminarily indicate that compared with β-disaccharide, the inductive mechanisms of lactose and cellulose carbon sources are similar to some extent. The functions of other predictive TFs have not been reported, and their roles in the transcriptional regulation of cellulase genes need further confirmation by experiments. Overall, these results describe a complicated system of transcription factors that modulate the gene expression of lignocellulase. These results offer a basis for further exploring new TFs related to cellulase synthesis and the induction mechanism of cellulase.

### Transporters

The mechanism by which fungi sense inducible sugars is still not fully understood, but researchers have suggested the participation of membrane proteins in this process (Havukainen et al. 2020; Zhang et al. 2013; Li et al. 2017a, b). Although the biology of this organism has been uncovered from multiple aspects, the functions of sugar transporters coded in its genome are unknown, with some exceptions. Approximately 5% (459 genes) of the *T. reesei* genome consists of genes that encode transporters. Among these genes, 46 were upregulated by MGD, and 42 were downregulated (*P*adj < 0.05 and log_2_-fold change > 1 or < -1; Additional file 9: Table S7).

The expression of transporter-encoding genes in *T. reesei* Rut C30 already characterized as sugar transporters or sensors is shown in Table 4. The cellulose sensor *Crt1* (TrireC30_109243), which is the putative β-disaccharide transporter, is reportedly important for cellulase induction (Havukainen et al. 2020), but there was no change in gene expression using MGD or lactose as the inducer. For the glucose/cellobiose transporter *Stp1* (TrireC30_136988) and glucose transporter *Hxt1* (TrireC30_124396) in *T. reesei* (Zhang et al. 2013; Ramos et al. 2006), the *Hxt1* gene is downregulated, but the *Stp1* gene is upregulated with MGD as an inducer. According to previous studies, the glucose low- and high-affinity transport system is conserved in microbes to handle natural variations in environmental nutrient accessibility (Li et al. 2021; Wang et al. 2017a, b). Because a high concentration of glucose exists in MGD, we speculate that *Hxt1* and *Stp1* prefer high- and low-affinity glucose transport systems, respectively. Interestingly, the two newly described putative lactose transporters (TrireC30_137795 and 137,001) are highly expressed in MGD (Porciuncula et al. 2013), but the expression of putative cellodextrin transporters (TrireC30_91594) was significantly reduced by sixfold. Previous studies have shown that transporters involved in lactose uptake could be induced by inducers of cellulase, including cellobiose and sophorose (Morikawa et al. 1995; Porciuncula et al. 2013). Therefore, it is possible that cellodextrin transporters may be involved in lactose transport. These cellodextrin transporters seem to be nonspecific, and TrireC30_26932, 124396 and 91594 are more likely to transport lactose, as mentioned above; however, TrireC30_137795 and TrireC30_137001 have a stronger affinity for β-disaccharide. Transcriptome analysis of the *T. reesei* and ∆*xyr1* strains shows that TF of *Xyr1* inhibits the gene expression of cupric ion transporter *Ctr* (TrireC30_139174) and zinc ion transporter (TrireC30_99586). The transcription of *Ctr* is downregulated, which is consistent with *Xyr1* being upregulated in MGD (Castro et al. 2016).

**Table 4:** Log_2_-fold change (FC) of characterized transporters in *T. reesei*

Gene ID	Name	log_2_FC	Up/down	Description
124396	Hxt1	− 5.63	Down	Glucose transporter
109243	Crt1	NS		Sensor
136988	Stp1	2.44	UP	Glucose/cellodextrin transporter
137229		NS		Fucose permease
137795		1.46	UP	Cellodextrin transporter
94416		− 1.34	Down	H^+^ sugar transporter
131359		− 2.84	Down	Toxin efflux pump
137001		0.96	UP	Cellodextrin transporter
91594		− 3.47	Down	Cellodextrin transporter
79984		NS		Cellodextrin transporter
139174	Ctr	− 0.96	Down	Copper transporter
99586		NS		Zinc transporter

NS, nonsignificant at *P*adj > 0.05

**Fig. 4:**
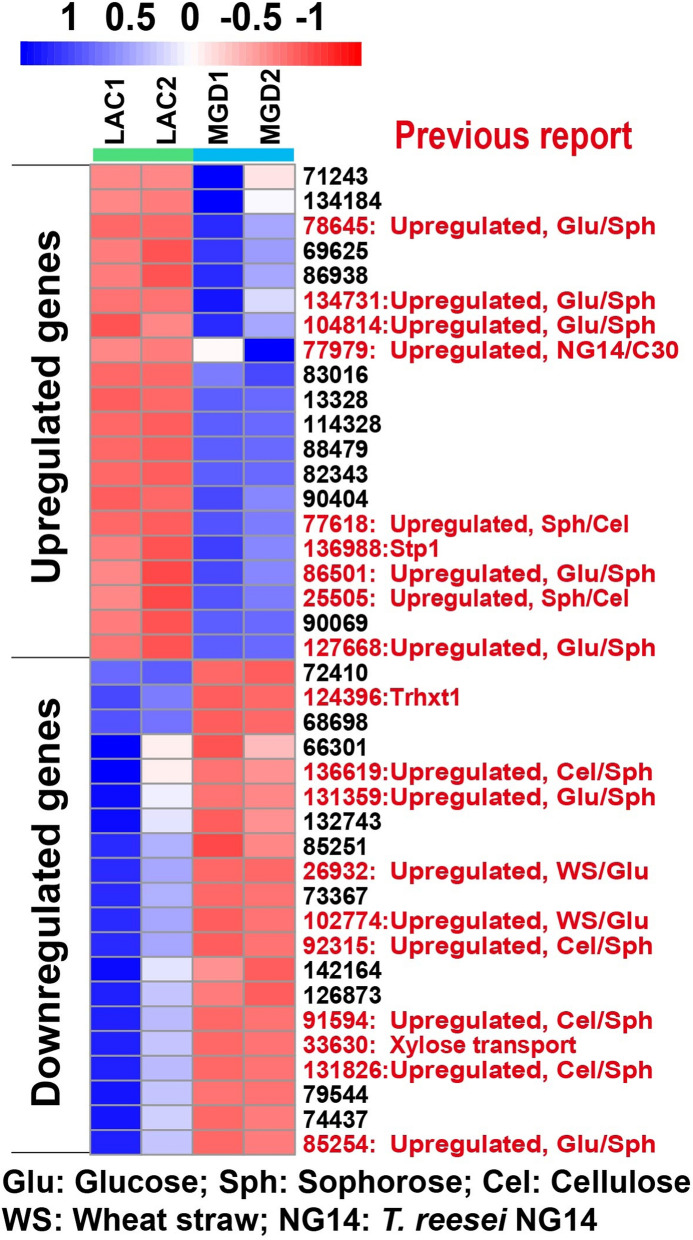
The top 40 genes of transporters that are differentially expressed in MGD or lactose. The columns for LAC1 and LAC2 show the gene expression data using 10 g/L lactose as an inducer at 48 h. The columns for MGD1 and MGD2 show the gene expression data using 10 g/L MGD as an inducer at 36 h. Data are shown as a heat map with the following color code of relative expression values (navy: 1; white: 0; firebrick: − 1; numbers indicate the log_2_ of the relative expression level). The previously reported transporters were explained

The top 40 transporter genes with significant differences in MGD/lactose were further analyzed (Fig. 4). Previous studies show that the genes TrireC30_78645, 134731, 104814, 86501 and 127668 are upregulated under glucose culture conditions compared to sophorose, which is displayed in red font (Castro et al. 2014). However, these genes are all involved in growth and metabolism instead of being sugar transporters. As reported, the TrireC30_77618 gene, predicted as a sugar transporter, is significantly upregulated with sophorose as an inducer compared to cellulose, which is similar to our present study. The other two transporters (TrireC30_25505 and 77979) are not considered to be sugar transporters related to metabolism (Castro et al. 2014).

Among the 20 specifically downregulated transporters in MGD, the gene encoding TrireC30_33630 has been proven to be involved in the transport of xylose (Saloheimo et al. 2007). A previous study showed that the genes TrireC30_136619, 92315 and 91594 were downregulated under sophorose culture conditions compared to cellulose, and our finding in MGD/lactose is consistent with this result (Castro et al. 2014). Combined with the above analysis of the GH family, we think these four transport pathways are potential sensors for the induction of xylanase because the activity of xylanase induced by MGD is lower than that of lactose or cellulose. The transporters in black font in Fig. 4 are reported for the first time, and BLASTP analysis of the sequences of these transporters did not reveal them to be sugar transporters, which is related to the differences in metabolism between MGD and lactose. Among the other downregulated genes, the transporter gene encoding the MFS permease (TrireC30_85251 and 74,437), which is largely upregulated in lactose, was predicted to be involved in the transport of β-disaccharides.

### MAPK pathway

The transduction of the signal is pivotal in cellulase production in *T. reesei*. At present, the signaling pathways of cellulase synthesis are focused on the MAPK and casein kinase II (CKII) pathways. The major TFs *Xyr1*, *Cre1*, *Ace1* and *Ace2* have MAPK phosphorylation sites (Wang et al. 2017a, b), and when the CKII phosphorylation sites of *Cre1* are destroyed, the feedback inhibition of carbon catabolite repression of *T. reesei* is removed (Cziferszky et al. 2002). However, if the *Cre1* structure of *T. reesei* Rut C30 is mutated, the MAPK cascades will be followed. The MAPK pathway is one of the most popular signal transduction systems. The corresponding signaling cascades consist of three serine/threonine kinases that function in series: MAPK kinase kinase, MAPK kinase and MAPK (Schmoll 2008).

**Fig. 5:**
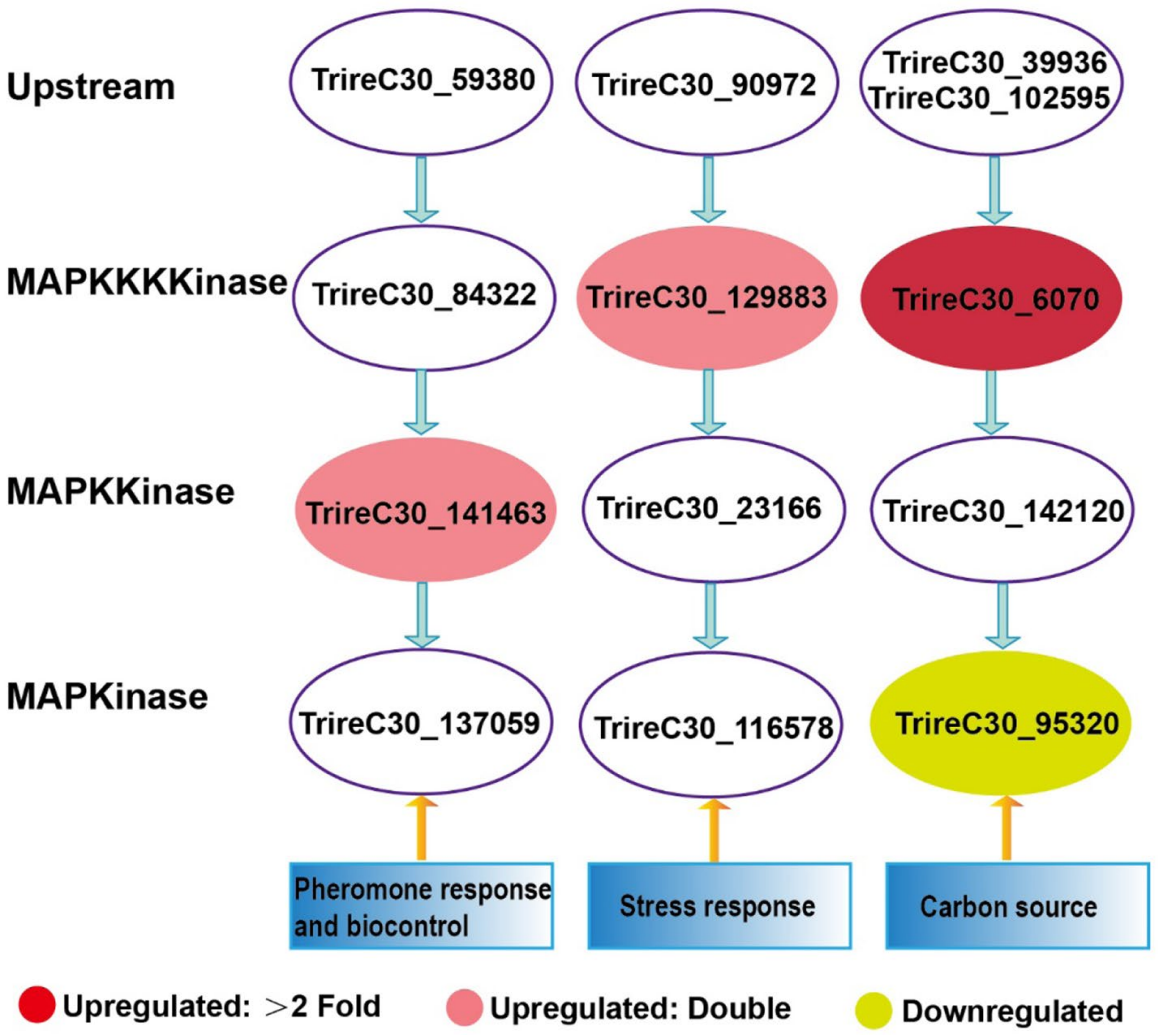
Differential transcription of genes involved in the MAP kinase cascade pathway, as deduced from reported functions and interactions of the respective nearest neighbors in *S. cerevisiae* and other filamentous ascomycetes according to Schmoll (2008)

The genes of the MAPK cascades showed different expression patterns when MGD or lactose is used as an inducer (Fig. 5). Four genes showed differences in expression levels: TrireC30_141463, TrireC30_129383 and TrireC30_6070 were significantly upregulated, while TrireC30_95320 was significantly downregulated in MGD compared to lactose. TrireC30_141463 is homologous to *S. cerevisiae Ste7p*, which is the MAPK of the pheromone response and biocontrol. In addition, TrireC30_129383 is related to *S. cerevisiae Bck1p*, which is a target for phosphorylation by protein kinase C (TrireC30_90792) in response to stress and protein degradation (Schmoll 2008). These two pathways are involved in the cell growth and cell wall integrity of *T. reesei* (Wang et al. 2017a, b), and they both participate in suppressing cellulase secretion, but they do not alter the transcription of cellulase-coding genes (Chen et al. 2015). The genes TrireC30_6070 and TrireC30_95320 (TrireC30_6070 is located upstream of TrireC30_95320) are involved in the MAPK response to osmosis and potentially carbon source sensing and positively regulate the expression of cellulase-coding genes. However, TrireC30_6070 and TrireC30_95320 were upregulated and downregulated, respectively (Fig. 5). The seemingly contradictory regulation phenomenon precisely reflects the coordination of cellular metabolism. Hence, the regulation of the MAPK pathway will become an effective method for further increasing the production of cellulase when MGD is used as an inducer.

### Endoplasmic reticulum (ER) protein processing pathway

**Fig. 6:**
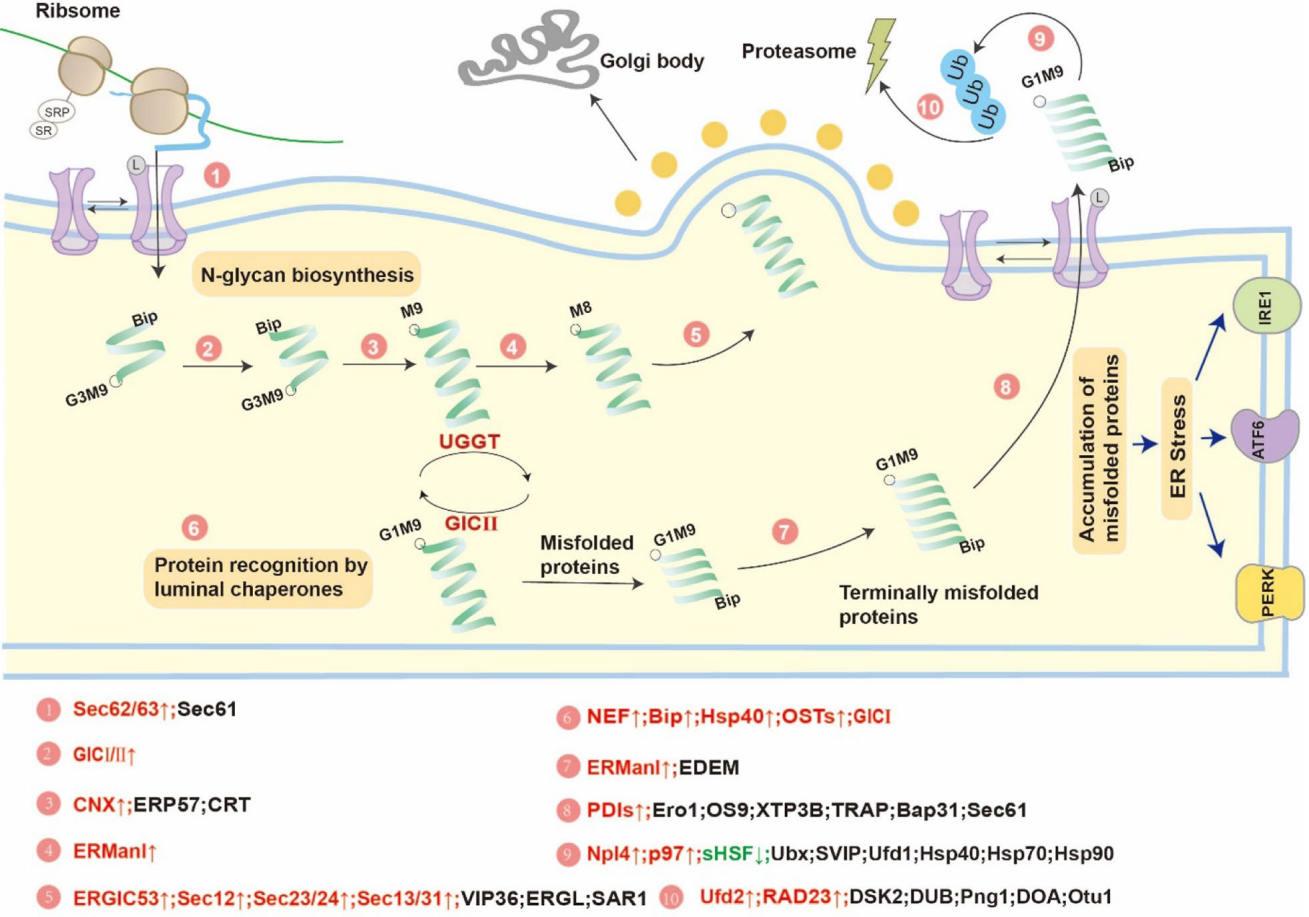
Differential transcription of genes involved in protein processing in the endoplasmic reticulum (ER). With the KEGG annotation result for ER, we also performed pathway functional enrichment using phyper, a function of R. Then, we calculated the false discovery rate (FDR) for each *p*-value; in general, the terms for which FDR was not larger than 0.05 were defined as significantly enriched

For filamentous fungi, peptides that enter the ER complete the signal peptide removal, glycosylation and correct protein folding with the participation of molecular chaperones. Our previous study showed that *T. reesei* has a higher protein production capacity when cultured in MGD compared to lactose (Li et al. 2016). Hence, quality control in the secretory pathway is a particular challenge since unusual polypeptides are identified and returned to the cytoplasm for proteasomal decomposition. The transcription of genes related to folding and processing of nascent proteins is depicted in Fig. 6 and Additional file 10: Table S8.

Lignocellulose, a newly synthesized secretory enzyme, enters the ER through channel complex unfolding. In this process, the gene expression of *Sec62/63* (TrireC30_111204), *OSTs* (TrireC30_107533, 142450 and 93903) and *GluI* (TrireC30_98040), which are all key genes for entering the ER during protein processing, increased by 2.0–2.6 times with MGD as an inducer (Conti et al. 2015; Mohanty et al. 2020). In addition, *Hsp70*-related molecular chaperones connect to nascent polypeptides in the ER lumen and to the cytosolic zones of membrane proteins, and they assist in substrate folding and disposal if folding fails. This process is enhanced in MGD; for example, the expression levels of *Nef* (TrireC30_99698), *BiP* (TrireC30_25648) and *Hsp40* (TrireC30_103637), which are involved in protein recognition by chaperones, increase by 2.6–3.6 times (Maio et al. 2021). Other genes responsible for the correct folding of proteins were also highly expressed, including *Cnx* (TrireC30_135283), *GlcII* (TrireC30_136825 and 141131) and *Uggt* (TrireC30_68830). The correctly folded proteins are enclosed in Golgi-targeted vesicles. This process is also strengthened by improving the expression levels of corresponding genes, including *Ergic53* (TrireC30_139361), *Sec12* (TrireC30_99637), *Sec13/31* (TrireC30_105903 and 131122) and *Sec23/24* (TrireC30_98502, 136978, 98597 and 103910). Moreover, any misfolded protein will be corrected. The expression of *ERMan1* (TrireC30_24645, 6341 and 91275) and *PDIs* (TrireC30_32116 and 26666) also increased in this process (Fu et al. 2020). These results are consistent with the fermentation behavior of *T. reesei* induced by MGD in which more proteins, mainly lignocellulase, enter the ER for correct folding and are released outside the cells.

Abnormal polypeptides, upon recognition, will be returned to the cytoplasm for proteasomal decomposition by ER-associated degradation. The expression of some genes in this process is improved, including *P97* (TrireC30_142542), *Npl4* (TrireC30_81981), *Rad23* (TrireC30_73603) and *Ufd2* (TrireC30_99049) (Dobson 2003). When unfolded proteins accumulate in the ER, resident protein chaperones of the ER will be occupied. In response to the accumulation of unfolded proteins in the lumen (ER stress), the ER activates signal transduction pathways in cells, which are collectively named the unfolded protein response (UPR) (Smyrnias 2021). In summary, at least three mechanistically different arms (ERK, IRE1 and ATF6) of the UPR modulate the expression of abundant genes that work in the secretory pathway and affect cell fate and the metabolism of proteins, amino acids and lipids in various ways. Excessive ER stress did not occur when MGD was used as an inducer, which is beneficial for the survival of *T. reesei*.

### RT-qPCR

**Fig. 7:**
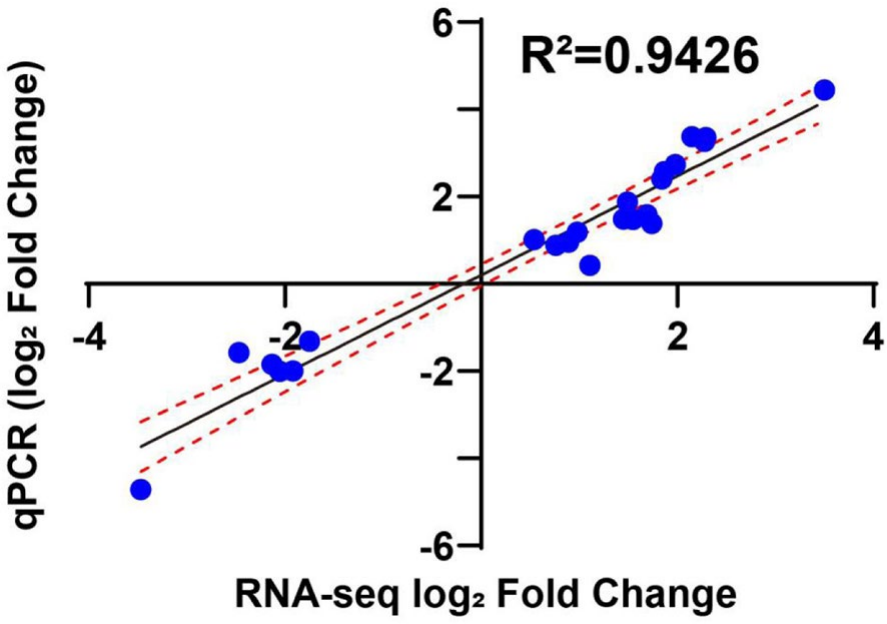
Correlation between RNA-seq and RT-qPCR. Comparison of the log_2_-fold change of 22 genes obtained by RNA-seq and RT-qPCR. RT-qPCR was performed using the amplified cDNA from each RNA-seq sample. A strong, statistically significant Pearson correlation is observed between the expression levels measured using RT-qPCR and RNA-seq

The transcriptome data were validated using RT-qPCR by detecting the mRNA expression levels of 23 genes in both MGD and lactose (Fig. 7). The 17 upregulated genes, mainly glycoside hydrolases, and 6 downregulated genes were randomly selected. The log_2_-fold change in gene expression among the three comparisons acquired from transcriptome and RT-qPCR implies a significant Pearson correlation (*R*^2^ = 0.9426), which indicates that the transcriptome analysis is reliable.

## Conclusions

This research provides the first transcriptomic analysis of *T. reesei* Rut C30 induced by a mixture of glucose and β-disaccharides (MGD). Both gene expression and the secretome are largely different in *T. reesei* grown in MGD or lactose. Our recent study showed that MGD can provoke higher transcription levels of major cellulases of *T. reesei* than lactose, but xylanase is deficient. Two transcription factors, *Vib1* and *Xyr1*, may play an important role in the regulation process. Some of the genes (TrireC30_6070, *Sec62/63*, *OSTs*, *GluI*, *Nef*, *BiP*, *Hsp40*, *Cnx*, *GlcII*, *Uggt*, etc.) in the MAPK pathway and ER protein processing pathway were also increased in MGD, which might serve as potential targets for further improving lignocellulase secretion in *T. reesei*. In addition, the present study also showed that TrireC30_137795 and TrireC30_137001 have stronger affinity for β-disaccharide and discovered potential lactose transporters (TrireC30_26932, 124396 and 91594). Overall, our data deepen the understanding of the utilization of MGD with *T. reesei*, which will promote the establishment of industrial strains of both *T. reesei* and yeast that generate abundant lignocellulase for plant cell wall degradation, thus contributing to its utilization in cellulosic ethanol generation.

### Supplementary Information

**Additional file 1: Fig S1.** Time-course of the batch culture of *T. reesei* Rut C30 on 10 g/L MGD or 10 g/L lactose. (A) Biomass, (B) Reducing sugars and (C) Protein.

**Additional file 2: Fig. S2.** Correlation between samples and the distribution of gene expression of RNA-seq data. (A) Heatmap of Pearson correlation between samples, (B) Gene expression Box-plot and (C) Gene expression density map.

**Additional file 3: Table S1.** Primers for qPCR analysis of the main cellulase- and UPR-related genes.

**Additional file 4: Table S2.** Clean reads quality metrics.

**Additional file 5: Table S3.** Summary of Genome Mapping.

**Additional file 6: Table S4.** The differentially expressed genes induced in the presence of MGD/lactose.

**Additional file 7: Table S5.** All of the carbohydrate active enzyme (CAZy) genes when incubated in MGD or lactose.

**Additional file 8: Table S6.** Up-/downregulated transcription factor genes in the presence of MGD/lactose.

**Additional file 9: Table S7.** Up-/downregulated transporter genes in the presence of MGD/lactose.

**Additional file 10: Table S8.** Upregulated genes related to the folding and processing of nascent proteins in the presence of MGD/lactose.

## Data Availability

The data presented in this study are deposited in the NCBI. The website link: https://www.ncbi.nlm.nih.gov/sra/PRJNA714230.

## Abbreviations

MGD: The mixture of glucose and β-disaccharide
*T. reesei*: *Trichoderma reesei*
TF: Transcription factor
AA: Auxiliary activity
SWO: Swollenin
LPMOs: Lytic polysaccharide monooxygenases
GH: Glycoside hydrolases
GO: Gene Ontology
KOG: EuKaryotic Orthologous Groups
NCBI: National Center for Biotechnology Information
UPR: Unfolded protein response
