# Supplementary figures and images for: Comparative transcriptome analysis of Trichoderma reesei reveals different gene regulatory networks induced by synthetic mixtures of glucose and β-disaccharide
Source: Bioresour Bioprocess. 2021 Jul 3;8(1):57. doi: 10.1186/s40643-021-00411-4 (PMC10991369; doi:10.1186/s40643-021-00411-4)

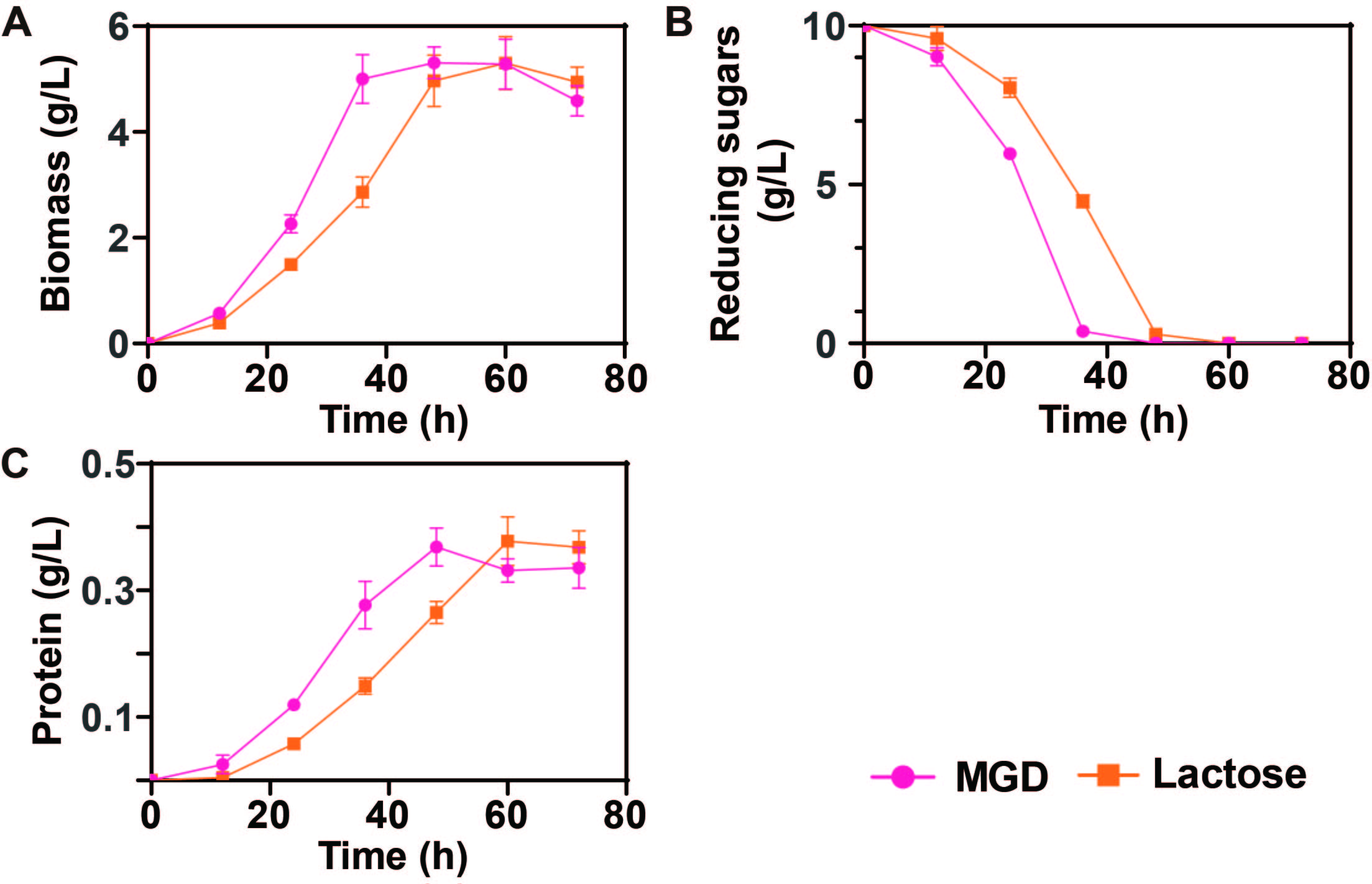

Supplement: Supplementary file 1 — Additional file 1: Fig S1. Time-course of the batch culture of T. reesei Rut C30 on 10 g/L MGD or 10 g/L lactose. (A) Biomass, (B) Reducing sugars and (C) Protein. [file 40643_2021_411_MOESM1_ESM.jpg]

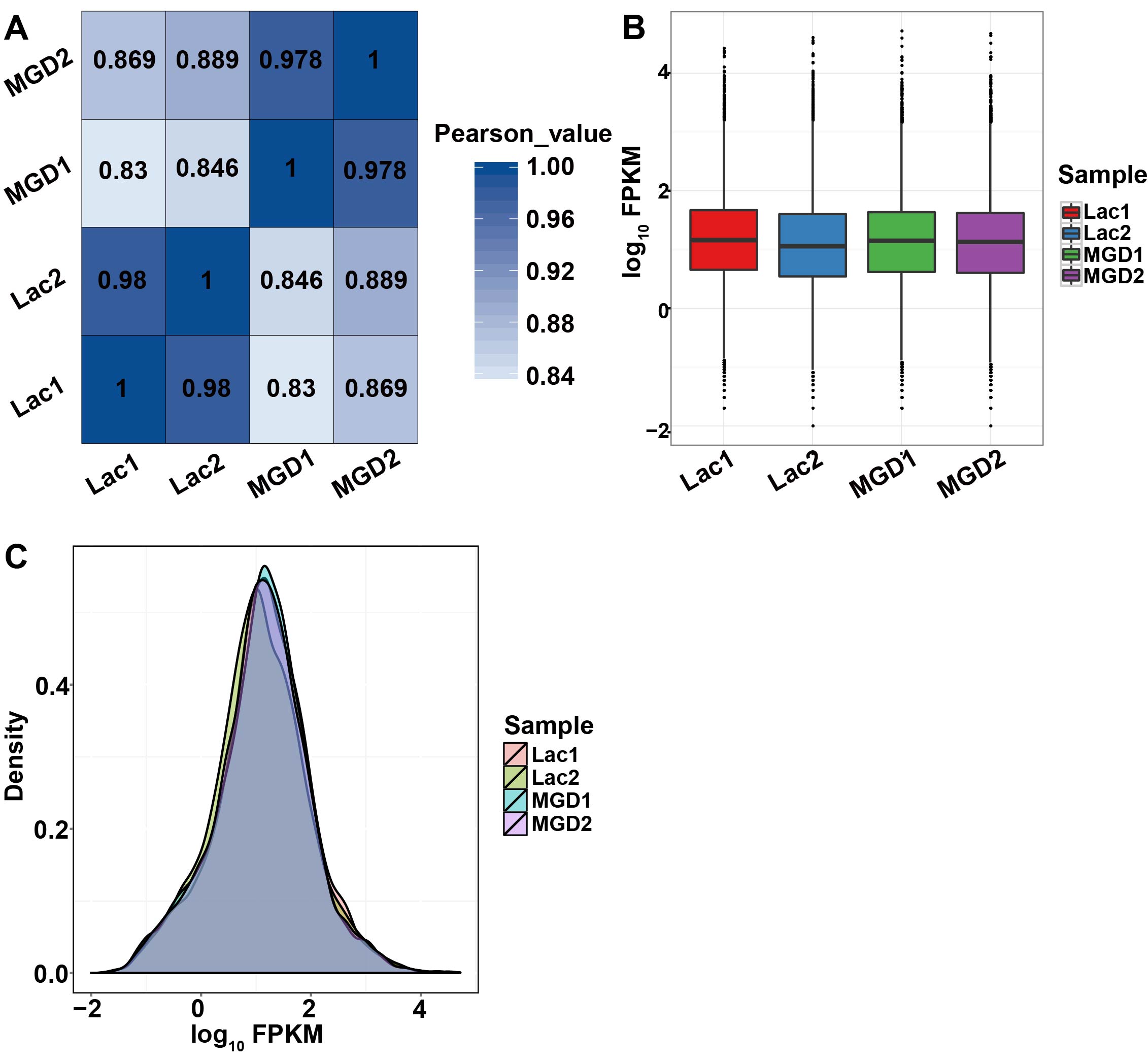

Supplement: Supplementary file 2 — Additional file 2: Fig. S2. Correlation between samples and the distribution of gene expression of RNA-seq data. (A) Heatmap of Pearson correlation between samples, (B) Gene expression Box-plot and (C) Gene expression density map. [file 40643_2021_411_MOESM2_ESM.jpg]
