# Supplementary material for: Comparative transcriptome analysis of Trichoderma reesei reveals different gene regulatory networks induced by synthetic mixtures of glucose and β-disaccharide
Source: Bioresour Bioprocess. 2021 Jul 3;8(1):57. doi: 10.1186/s40643-021-00411-4 (PMC10991369; doi:10.1186/s40643-021-00411-4)
Supplement: Supplementary file 3 — Additional file 3: Table S1. Primers for qPCR analysis of the main cellulase- and UPR-related genes. [file 40643_2021_411_MOESM3_ESM.docx]

Table S1 Primers for qPCR analysis of the main cellulase and UPR−related genes

| Primer | | Sequence (5’−3’) |
| --- | --- | --- |
| *cbh1* | F | ACGAGTTCTCTTTCGATGTTGATG |
|  | R | CGGTGTTGGTGGGATACTTG |
| *egl1* | F | CTGGTGGCTAGTGTTGAGGG |
|  | R | CCGAGTGATCTGTTCCAGAATGT |
| *xyn1* | F | TGGCTCTGCTTCCATCACC |
|  | R | CCCCTTTTATCCACCATCCTTT |
| *swo1* | F | GCTTCCACCTACACAACCACA |
|  | R | TGGGCAGCAAACATTATCCA |
| *eg4* | F | CAATGGTGACTGCGAGACC |
|  | R | CCAGGTGTTGTTGTTGGAGA |
| *ire1* | F | CTGAGCCGGAGCTAAAGTTG |
|  | R | ATGACTACGCCAGCAAGGAC |
| *hac1* | F | ATCTTCCGTCTTTGCACGTT |
|  | R | CTACCGCACCCTCTACCTCA |
| *pdi1* | F | TCGCTCTTGGCATACAGG |
|  | R | CGTTGTCGTTGCCCACT |
| *bip1* | F | TTGTCGTTGGTGATGGTGAT |
|  | R | CCAGATTGAGGTTTCCTTCG |
| *101839* | F | CGACTTCCACTCCACTATG |
|  | R | CCTCTTCTCCGTGTTGTC |
| *92206* | F | CTCTTCTTCTTCTTCTTCATCATC |
|  | R | GCGACGACATTGAGATAGT |
| *93861* | F | CTGGCGTACTATGATGTCTT |
|  | R | GCTGTAGGCGTCTATGTC |
| *31634* | F | CTTCACTTTCGGCTCCTT |
|  | R | GCACATTCTGGCACATTC |
| *96282* | F | CTGCCTACCATTACGGAAT |
|  | R | CTCCTCTTCGCTCTTGAC |
| *125610* | F | CGACGGCTCCTATTGTAG |
|  | R | ATGTCCACACTTCTCTTGAG |
| *90707* | F | GCTTGGAGATGTCAGTTGA |
|  | R | TTGTTGTTGCTGCGATTG |
| *128051* | F | TATCGTTACTGGAACAAGACATA |
|  | R | AAGGTCGGCTATCATAAGTG |
| *59591* | F | CACTCAGCGAAGCATACT |
|  | R | CATATAGGCGGCATCTCTC |
| *79514* | F | GGAGGAATGAGCACTTGAA |
|  | R | TGGTTGTTGAGGTTGTCTT |
| *77618* | F | TGGAGTGGCTTATCTGATTC |
|  | R | GGATATACTTCTGATGGGACAA |
| *25505* | F | CTGGCGACTCTGAAGAAG |
|  | R | AAGATGCTGATGCTGACAA |
| *91594* | F | GGTGTTGTCTTGGTTCCT |
|  | R | CAGATGTCGCAGAGTTGA |
| *136619* | F | CATTCCTTCTCTTCGGCTAT |
|  | R | ATGGTGGCATCTCTTGTC |
