# Supplementary material for: Comparative transcriptome analysis of Trichoderma reesei reveals different gene regulatory networks induced by synthetic mixtures of glucose and β-disaccharide
Source: Bioresour Bioprocess. 2021 Jul 3;8(1):57. doi: 10.1186/s40643-021-00411-4 (PMC10991369; doi:10.1186/s40643-021-00411-4)
Supplement: Supplementary file 4 — Additional file 4: Table S2. Clean reads quality metrics. [file 40643_2021_411_MOESM4_ESM.docx]

Table S2 Clean reads quality metrics

| Sample | Total Raw Reads (Mb) | Total Clean Reads (Mb) | Total Clean Bases (Gb) | Clean Reads Q20 (%) | Clean Reads Q30 (%) | Clean Reads Ratio (%) |
| --- | --- | --- | --- | --- | --- | --- |
| Lac1 | 40.6 | 30.34 | 4.55 | 96.78 | 90.81 | 74.72 |
| Lac2 | 39.19 | 29.64 | 4.45 | 96.88 | 91.11 | 75.63 |
| MGD1 | 40.83 | 30.06 | 4.51 | 96.71 | 90.73 | 73.63 |
| MGD2 | 38.23 | 28.45 | 4.27 | 96.73 | 90.56 | 74.41 |
