# Supplementary material for: Comparative transcriptome analysis of Trichoderma reesei reveals different gene regulatory networks induced by synthetic mixtures of glucose and β-disaccharide
Source: Bioresour Bioprocess. 2021 Jul 3;8(1):57. doi: 10.1186/s40643-021-00411-4 (PMC10991369; doi:10.1186/s40643-021-00411-4)
Supplement: Supplementary file 5 — Additional file 5: Table S3. Summary of Genome Mapping. [file 40643_2021_411_MOESM5_ESM.docx]

Table S3 Summary of Genome Mapping

| Sample | Total Clean Reads | Total Mapping Ratio | Uniquely Mapping Ratio |
| --- | --- | --- | --- |
| Lac1 | 30340762 | 91.77% | 75.10% |
| Lac2 | 29642788 | 91.77% | 74.46% |
| MGD1 | 30063128 | 91.25% | 72.28% |
| MGD2 | 28446574 | 91.82% | 72.91% |
